# CgCFEM1 Is Required for the Full Virulence of *Colletotrichum gloeosporioides*

**DOI:** 10.3390/ijms25052937

**Published:** 2024-03-02

**Authors:** Liping Feng, Meixia Dong, Zhirui Huang, Qian Wang, Bang An, Chaozu He, Qiannan Wang, Hongli Luo

**Affiliations:** 1 Sanya Nanfan Research Institute of Hainan University, School of Tropical Agriculture and Forestry, Hainan University, Sanya 572025, China; 2 Hainan Yazhou Bay Seed Laboratory, Sanya 572025, China

**Keywords:** *Colletotrichum gloeosporioides*, CgCFEM1, appressorium morphogenesis, plant immunity

## Abstract

*Colletotrichum gloeosporioides* is widely distributed and causes anthracnose on many crops, resulting in serious economic losses. Common fungal extracellular membrane (CFEM) domain proteins have been implicated in virulence and their interaction with the host plant, but their roles in *C. gloeosporioides* are still unknown. In this study, a CFEM-containing protein of *C. gloeosporioides* was identified and named as CgCFEM1. The expression levels of *CgCFEM1* were found to be markedly higher in appressoria, and this elevated expression was particularly pronounced during the initial stages of infection in the rubber tree. Absence of CgCFEM1 resulted in impaired pathogenicity, accompanied by notable perturbations in spore morphogenesis, conidiation, appressorium development and primary invasion. During the process of appressorium development, the absence of CgCFEM1 enhanced the mitotic activity in both conidia and germ tubes, as well as compromised conidia autophagy. Rapamycin was found to basically restore the appressorium formation, and the activity of target of rapamycin (TOR) kinase was significantly induced in the *CgCFEM1* knockout mutant (∆*CgCFEM1*). Furthermore, CgCFEM1 was proved to suppress chitin-triggered reactive oxygen species (ROS) accumulation and change the expression patterns of defense-related genes. Collectively, we identified a fungal effector CgCFEM1 that contributed to pathogenicity by regulating TOR-mediated conidia and appressorium morphogenesis of *C. gloeosporioides* and inhibiting the defense responses of the rubber tree.

## 1. Introduction

The intricate molecular interactions between plant and fungal pathogen underlie the development of many plant diseases [1]. In the established paradigm of plant–pathogen interactions, when a pathogen lands on a plant, the conserved microbial molecular signatures, termed pathogen- or microbe-associated molecular patterns (PAMPs) were recognized by cell-surface pattern recognition receptors (PRRs) in plants to activate pattern-triggered immunity (PTI). To overcome PTI, the pathogen secreted effector molecules to evade or suppress PTI, which resulted in effector-triggered susceptibility (ETS) [2,3].

Common in the fungal extracellular membrane (CFEM) domain, it is fungal-specific and consists of about 60 amino acid residues containing 8 spaced cysteine residues with the consensus sequence PxC [A/G] x2Cx8-12Cx1-3[x/T] Dx2-5CxCx9-14Cx3-4Cx15-16 (x represents any residue with its range shown) [4,5]. Depending on the presence of transmembrane domains, fungal CFEM-domain-containing proteins were classified into two distinct types: Pth11-like type and non-Pth11-like type. Pth11-like CFEM proteins, represented by Pth11, which was an essential factor for pathogenesis in the rice blast fungus, comprised multiple transmembrane domains and functioned as extracellular receptors, signal transducers, or adhesion molecules in host–pathogen interactions [6,7,8,9,10]. Conversely, non-Pth11-like CFEM proteins were presumed secreted proteins containing signal peptide sequences, yet devoid of transmembrane domains, and acted as an effector which manipulated plant immunity [9,10]. While the general length of CFEM domains remains conserved, the quantity of CFEM domains varies extensively among diverse CFEM-domain-containing proteins. Intriguingly, pathogenic fungi tend to possess more copies of CFEM-domain-containing proteins, featuring an augmented number of CFEM domains compared to their non-pathogenic counterparts [5], indicating the potential significant roles that CFEM-domain-containing proteins might play in fungal virulence.

Pth11-like type CFEM proteins of phytopathogenic fungi appeared to impact pathogenicity through their regulation of growth, development, and the formation of invasive structures [4,6,7,11,12,13,14]. Non-Pth11-like type CFEM proteins played a role in driving the virulence of phytopathogenic fungi and their interactions with host plants [9,15,16,17,18]. In *Botrytis cinerea*, a non-Pth11-like type CFEM protein BcCFEM1with a signal peptide and a Glycosylphosphatidylinositol (GPI)-anchored site, contributed to virulence, conidial production and stress tolerance [15]. In *Verticillium dahliae*, VdSCP76 and VdSCP77, two non-Pth11-like type CFEM proteins with signal peptide but lacking GPI-anchored site, played critical roles in virulence on cotton plants through the suppression of immunity [17]. Nevertheless, not all CFEM proteins contributed to fungal virulence. For instance, three *Aspergillus fumigatus* CFEM-motif GPI-anchored proteins CfmA-C influence cell wall stability without demonstrable effects on fungal virulence [19]. Taken together, the collective findings from these studies fully indicated the functional diversity of CFEM proteins in plant pathogenic fungi.

*Colletotrichum* is one of the most common and important genera of plant pathogenic fungi causing anthracnose in over 3200 monocot and dicot plant species [20,21]. *Colletotrichum* species usually used a multistage hemibiotrophic infection strategy including penetration, growth inside living host cells (biotrophic stage) and tissue destruction (necrotrophic stage). Multiple effectors, including CFEM proteins, were involved in both stages and the regulation of the stage switch [21,22,23]. In *Colletotrichum graminicola*, 24 CgCFEM proteins were identified, with 10 of them considered as effectors with a signal peptide and without the transmembrane domain, which had different subcellular localizations in host cells and might play important roles during the pathogenic processes on maize plants [8]. *C. gloeosporioides* was widely distributed in tropical, subtropical, and temperate regions and infected woody plants, resulting in serious economic losses annually [24]. Natural rubber is an irreplaceable, important industrial raw material and mainly produced from the rubber tree (*Hevea brasiliensis*). Rubber tree anthracnose caused by *C. gloeosporioides* resulted in serious loss of natural rubber production worldwide [25]. At present, little is known about the pathogenesis mediated by the CFEM-containing effector of *C. gloeosporioides* on the rubber tree. In this study, a candidate CFEM-containing effector CgCFEM1 of *C. gloeosporioides* was identified and its contribution to fungal virulence and development was investigated. These results will provide new insights into the pathogenicity of *C. gloeosporioides* to rubber trees.

## 2. Results

### 2.1. CgCFEM1 Contained a Signal Peptide and a Conserved CFEM Domain

**Figure 1:**
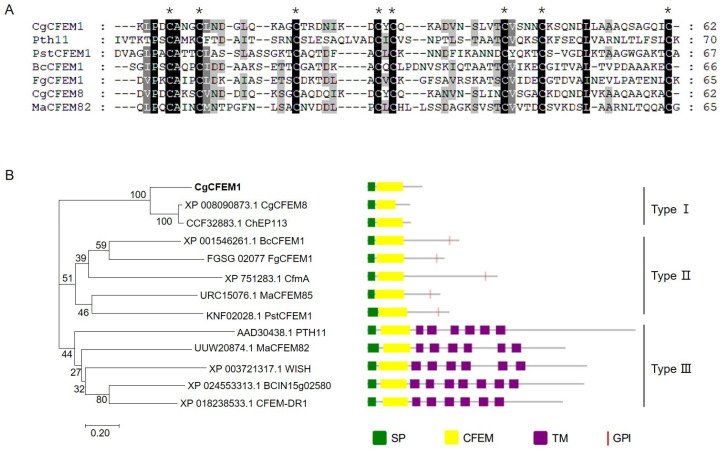
Multiple sequence alignment and phylogenetic analysis of CgCFEM1. (**A**) Alignment of CFEM domains from the CFEM proteins of different microorganisms. Conserved amino acids are highlighted in black and gray, 8 conserved cysteine residues are labeled with asterisks. The CFEM domains used for alignment were Pth11 from *Magnaporthe oryzae* (AAD30438.1), PstCFEM1 from *Puccinia striiformis* f. sp. *tritici* (KNF02028.1), BcCFEM1 from *Botrytis cinerea* (XP_001546261.1), FgCFEM1 from *Fusarium graminearum* (FGSG_02077), CgCFEM8 from *Colletotrichum graminicola* (XP_008090873.1), and MaCFEM82 from *Metarhizium anisopliae* (UUW20874.1). (**B**) Phylogenetic tree of CgCFEM1 with different types of CFEM proteins in fungi. The green box represents the length and localization of the signal peptide, the yellow box represents the CFEM domain, the purple box represents the presence of a transmembrane domain (TM), and the red vertical line represents the C-terminal potential site for glycosylphosphatidylinositol (GPI) modification. BcCFEM1 and BCIN15g02580 (XP_024553313.1) are from *B. cinerea*, CFEM-DR1 (XP_018238533.1) is from *Fusarium oxysporum*, CfmA (XP_751283.1) is from *Aspergillus fumigatus*, CgCFEM8 is from *C.graminicola*, ChEP113 (CCF328831) is from *Colletotrichum higginsianum*, FgCFEM1 is from *F.graminearum*, MaCFEM82 and MaCFEM85 (URC15076.1) are from *M.anisopliae*, PstCFEM1 is from *P. striiformis* f. sp. *tritici*, Pth11 and WISH (XP_003721317.1) are from *M. oryzae*.

In order to investigate the pathogenic mechanism of *C. gloeosporioides* to the rubber tree, a gene encoding an extracellular secreted protein was predicted and named as *CgCFEM1* (OR394961). The encoding region of *CgCFEM1* contained 387 bp encoding a 128 aa protein with a signal peptide (1–18 aa) at its N-terminal and a CFEM domain (23–84 aa) (Supplementary Figure S1). The alignment of the CFEM domain in CgCFEM1 with that in some identified CFEM proteins from different fungi, including *Magnaporthe oryzae* Pth11(AAD30438.1), *Puccinia striiformis* f. sp. *tritici* PstCFEM1(KNF02028.1), *B*. *cinerea* BcCFEM1(XP_001546261.1), *Fusarium graminearum FgCFEM* (FGSG_02077), *C. graminicola* CgCFEM8 (XP_008090873.1), *Metarhizium anisopliae* MaCFEM82 (UUW20874.1), showed that CgCFEM1 contained a typical CFEM domain with spaced 8 cysteine residues with the consensus sequence PxCA x2Cx8-12Cx1-3[x/T]Dx2-5CxCx9-14Cx3-4Cx15-16 (x represents any residue with its range shown) (Figure 1A). The CFEM containing proteins identified from fungal species, including CgCFEM1, were used to generate a neighbor-joining tree (Figure 1B). Phylogenetic tree analysis showed that our CgCFEM1 was closely related to a CFEM protein of *C. graminicola* (CgCFEM8), which was the same genus different species with *C. gloeosporioides.* Moreover, CgCFEM1 was clustered in the branch of non-Pth11-like type CFEM proteins without GPI-anchored site, which was defined as type I CFEM proteins in this study. In addition, based on the architecture of phylogenetic tree, we defined non-Pth11-like type CFEM proteins with GPI-anchored site as Type II CFEM proteins and Pth11-like type CFEM proteins as Type III CFEM proteins (Figure 1B).

### 2.2. High-Level Expression of CgCFEM1 in Appressorium and Early Infection Stage

**Figure 2:**
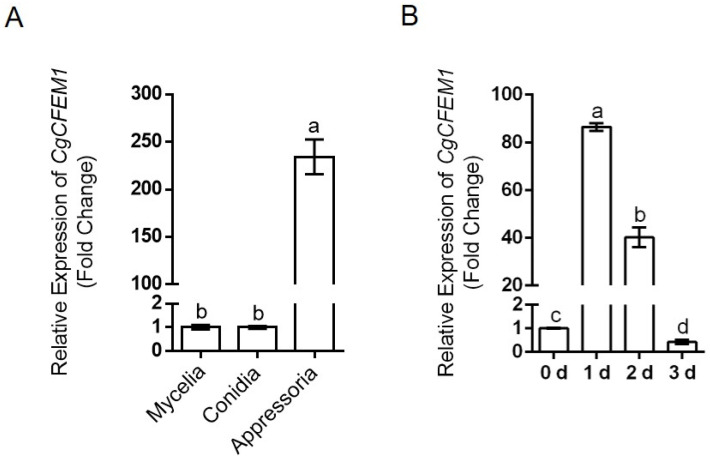
The transcript expression pattern of *CgCFEM1*. (**A**) The relative expression patterns of *CgCFEM1* at different development stages. (**B**) The relative expression pattern of *CgCFEM1* during infection on rubber tree leaves. Data of (**A**,**B**) are shown as the means ± standard deviation (SD) from three independent experiments, and columns with different letters indicate a significant difference (*p* < 0.05).

To explore the roles of *CgCFEM1* in the development of *C. gloeosporioides* and infection process towards the rubber tree, the expression levels of *CgCFEM1* were examined in mycelia, conidia, appressoria, and during leaf infection. The results showed that the expression level of *CgCFEM1* in mycelia and conidia was comparable, but that the one in appressoria was two hundred times more than that in mycelia and conidia (Figure 2A). Additionally, the expression level of *CgCFEM1* was induced more than 80-fold in rubber tree leaves at 1 d post inoculation with *C. gloeosporioides* and then gradually decreased to the original level at 3 d post inoculation (Figure 2B). These results suggested the potential role of *CgCFEM1* in regulating the pathogenicity of *C. gloeosporioides* towards rubber trees.

### 2.3. CgCFEM1 Contributed to Pathogenicity

**Figure 3:**
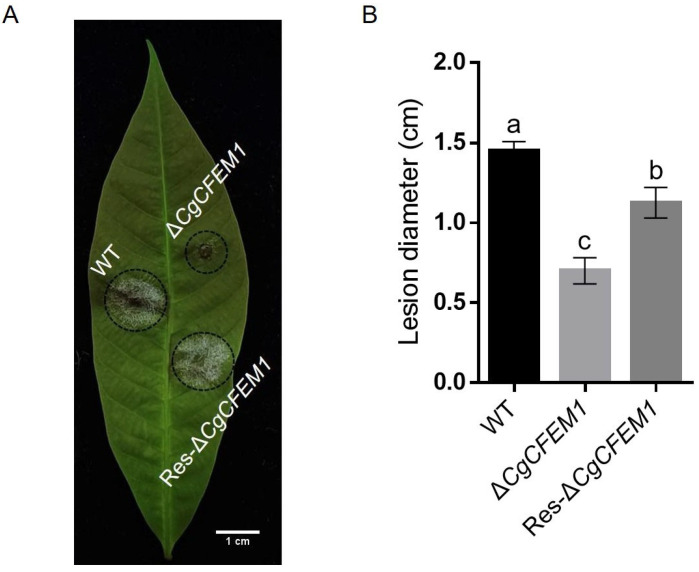
Pathogenicity assay of WT, Δ*CgCFEM1* and Res-Δ*CgCFEM1* on rubber tree leaves. (**A**) Disease symptoms of rubber tree leaves at 4 days post-inoculation (dpi) with WT, Δ*CgCFEM1* and Res-Δ*CgCFEM1*, respectively. Scale Bar = 1 cm. (**B**) Statistical analysis of lesion diameter after inoculation with WT, Δ*CgCFEM1* and Res-Δ*CgCFEM1*. Data are shown as the means ± SD from three independent experiments, and columns with different letters indicate a significant difference (*p* < 0.05).

The *CgCFEM1* knockout strains ∆*CgCFEM1* and complementary strains Res-∆*CgCFEM1* were generated for function assay by gene replacement and gene insertion through PEG-mediated protoplast transformation. The schematic diagrams of knockout mutants and complementary mutants were shown in Figure S2A,C. PCR detections were performed to verify the ∆*CgCFEM1* mutants and Res-∆*CgCFEM1* mutants (Figure S2B,D). The detached leaf inoculation assay showed that all the tested strains, including wild type (WT), ∆*CgCFEM1* and Res-∆*CgCFEM1*, caused typical necrotic lesions at 4 days post-inoculation (Figure 3A). Statistical analysis showed that the size of the necrotic lesions induced by ∆*CgCFEM1* were significantly smaller than those induced by WT and Res-∆*CgCFEM1*, and Res-∆*CgCFEM1* restored the virulence of ∆*CgCFEM1* (Figure 3B). These data indicated that *CgCFEM1* contributed to the pathogenicity of *C. gloeosporioides* to the rubber tree.

### 2.4. CgCFEM1 Contributed to Spore Morphogenesis, Conidiation, Appressorium Development and Primary Invasion

**Figure 4:**
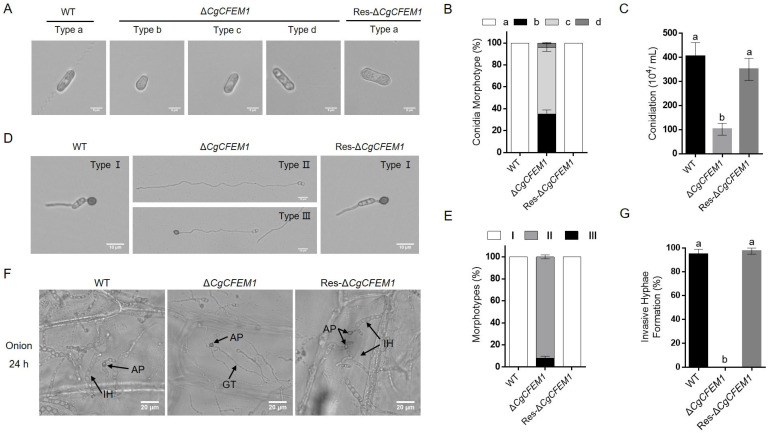
Conidia and appressoria differentiation of WT, Δ*CgCFEM1* and Res-Δ*CgCFEM1*. (**A**) Conidia morphotypes in WT, Δ*CgCFEM1* and Res-Δ*CgCFEM1*. Type a, normal conidia; Type b, near-spherical conidia; Type c, shorter than normal conidia; Type d longer than normal conidia. Scale Bar = 5 µm. (**B**) Statistical analysis of different conidia types in WT, Δ*CgCFEM1* and Res-Δ*CgCFEM1*. (**C**) Conidia production of WT, Δ*CgCFEM1* and Res-Δ*CgCFEM1* in CM media. (**D**) Types of appressoria differentiation and formation in WT, Δ*CgCFEM1* and Res-∆*CgCFEM1* at 12 h post incubation. Type I, normal germ tube with a mature appressorium; Type II, elongated germ tube with no appressoria; Type III, elongated germ tube with dysplastic appressoria. Scale Bar = 10 µm. (**E**) Statistical analysis of different types of appressorium differentiation and formation in WT, Δ*CgCFEM1* and Res-Δ*CgCFEM1* at 12 h post incubation. (**F**) Invasion observation in onion epidermal cells. Equal volumes (5 µL) of conidial suspensions (2.5 × 10^5^ conidia mL^−1^) from WT, Δ*CgCFEM1* and Res-Δ*CgCFEM1* were inoculated with the onion epidermal cells at 24 h post-inoculation (hpi). AP, IH, GT indicate the appressorium, invasive hyphae and germ tube, separately. Scale Bar = 20 µm. (**G**) Invasive hyphae formation rates of WT, Δ*CgCFEM1* and Res-Δ*CgCFEM1* at 24 hpi. Ten microscope fields with at least 100 conidia were surveyed for every sample. Different letters above columns indicate a significant difference (*p* < 0.05).

The growth rate of the colony, spore morphogenesis, conidiation ability, conidia germination and appressorium formation of ∆*CgCFEM1* were analyzed to assess the roles of CgCFEM1 in fungal growth and development. Although there was no significant difference in the colony growth rate between ∆*CgCFEM1* and WT (Figure S3A,B), the conidia morphology of ∆*CgCFEM1* showed diversity, with some near-spherical (Type b), some shorter (Type c) and some longer (Type d) compared to the normal conidia of WT and Res-∆*CgCFEM1* (Type a) (Figure 4A). In ∆*CgCFEM1*, type b and type c conidia accounted for about 35% and 60%, respectively, and type d conidia accounted for only about 5% (Figure 4B). The conidia production of ∆*CgCFEM1* was only a quarter of that of WT (Figure 4C). Furthermore, the process from conidia germination to the appressorium formation was analyzed in WT, ∆*CgCFEM1* and Res-∆*CgCFEM1*. In WT and Res-∆*CgCFEM1*, the conidia normally germinated and formed a mature appressorium at the tip of germ tubes (Type I) in 12 h post incubation. However, in ∆*CgCFEM1,* about 90% of conidia germinated to form significant longer germ tubes without an appressorium (Type II) and the remainder conidia germinated to form significant longer germ tubes with a dysplastic appressorium (Type III) (Figure 4D,E). In addition, the primary invasion of ∆*CgCFEM1* was tested on onion epidermis and was observed under an optical microscope. Compared to WT and Res-∆*CgCFEM1*, which could successfully complete infection and form normal primary invasive hyphae in onions epidermis cells, ∆*CgCFEM1* rarely formed primary hyphae in onion epidermis cells (Figure 4F,G). These data demonstrated that *CgCFEM1* played an important role in conidia morphogenesis and the development of the invasion structure of *C. gloeosporioides*.

### 2.5. CgCFEM1 Was Involved in the Regulation of Cell Cycle Progression in Conidia and Germ Tube

**Figure 5:**
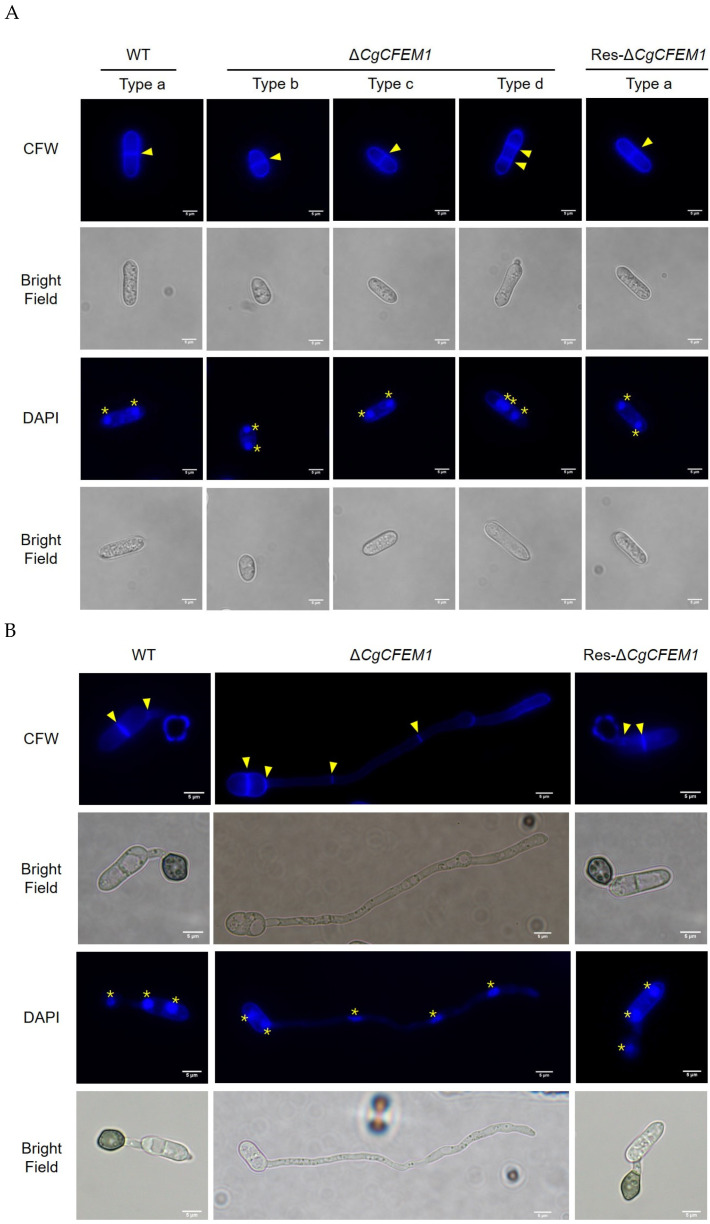
CFW and DAPI staining for conidia and germ tube of WT, Δ*CgCFEM1* and Res-Δ*CgCFEM1*. (**A**) CFW and DAPI staining for conidia of WT, Δ*CgCFEM1* and Res-Δ*CgCFEM1*. (**B**) CFW and DAPI staining for germ tubes of WT, Δ*CgCFEM1* and Res-Δ*CgCFEM1* at 8 h post incubation (hpi). CFW and DAPI were used to stain the septum and nuclei, respectively. Yellow triangles represent the septa. Yellow asterisks indicate the presence of the nucleus. Scale Bar = 5 µm.

As mentioned above, the length of conidia and the germ tube of ∆*CgCFEM1* were significantly different from that of WT (Figure 4A,D). To determine whether these changes of conidia and germ tube length were due to cell size or cell numbers, calcofluor white (CFW) and 2-(4-Amidinophenyl)-6-indolecarbamidine dihydrochloride (DAPI) staining were performed on conidia and its germ tubes. The results showed that Type b and Type c conidia from ∆*CgCFEM1* and Type a conidia from WT and Res-∆*CgCFEM1* had one septum and Type d conidia from ∆*CgCFEM1* had two septa (Figure 5A). The germ tube of ∆*CgCFEM1* mostly contained four septa, compared to the usual WT germ tube with two septa (Figure 5B). These data demonstrated that loss of CgCFEM1 resulted in multiple-round mitosis of the conidia and germ tube, indicating the involvement of CgCFEM1 in the regulation of cell cycle progression of the conidia and germ tube.

### 2.6. CgCFEM1 Was Involved in Conidia Autophagy during Appressorium Development

**Figure 6:**
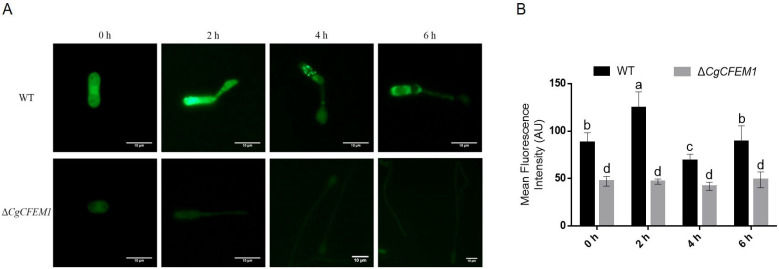
MDC staining for conidial autophagy in WT and ∆*CgCFEM1*. (**A**) Autophagic vacuoles were stained with 40 μM MDC and examined under a fluorescence microscope at 0 hpi, 2 hpi, 4 hpi and 6 hpi. Scale Bar = 10 µm. (**B**) Fluorescence intensity in WT and ∆*CgCFEM1* at the indicated time points. Different letters above columns indicate a significant difference (*p* < 0.05).

To investigate whether the regulation of CgCFEM1 on fungal appressorium development is related to autophagy, the conidial autophagy in ∆*CgCFEM1* during appressorium development was analyzed by monodansylcadaverine (MDC) staining. The results showed that autophagosomes were obviously observed in the conidia of wild type (WT) after appressorium formation induction, but not in the conidia of ∆*CgCFEM1* (Figure 6A). The fluorescence intensity in WT was also stronger than that in ∆*CgCFEM1* (Figure 6B). This data revealed that loss of CgCFEM1 impaired conidia autophagy during appressorium development, indicating the involvement of CgCFEM1 in autophagy related with appressorium development.

### 2.7. CgCFEM1 Contributed to Appressorium Formation through TOR Signaling

**Figure 7:**
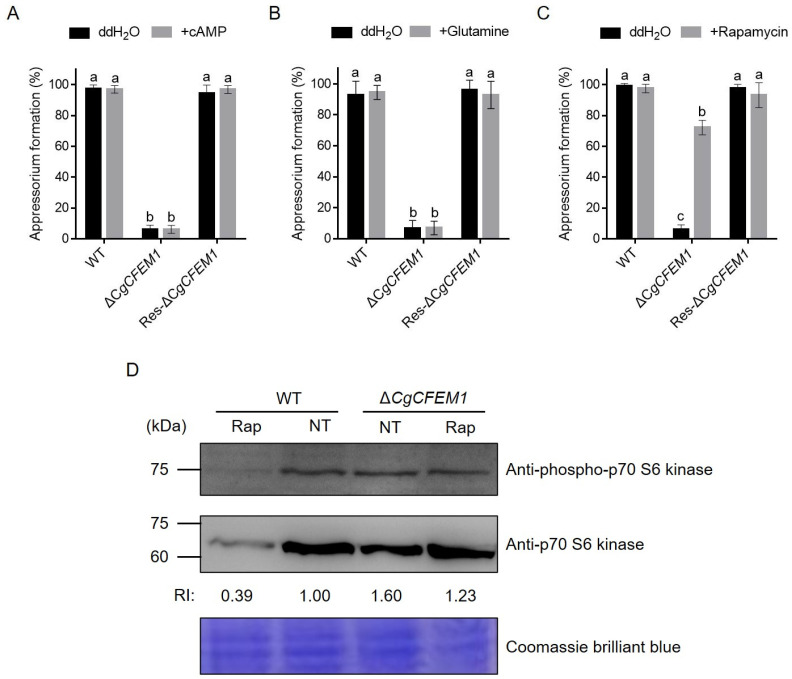
Effects of cAMP, glutamine and rapamycin on the appressorium formation rates of WT, Δ*CgCFEM1* and Res-Δ*CgCFEM1*. (**A**) Appressorium formation rates of WT, ΔCg*CFEM1* and Res-Δ*CgCFEM1* at 24 hpi with 10 mM 8-bromoadenosine 3′,5′ -cyclic monophosphate sodium salt (8-Br-cAMP). Different letters above columns indicate a significant difference (*p* < 0.05). (**B**) Appressorium formation rates of WT, ΔCg*CFEM1* and Res-Δ*CgCFEM1* at 24 hpi with 10 mM L-glutamine. (**C**) Appressorium formation rates of WT, ΔCg*CFEM1* and Res-Δ*CgCFEM1* at 24 hpi with 100 nM rapamycin. (**D**) Immunoblot showing the phosphorylation status of the direct TOR kinase target Sch9 in the indicated strains following treatment with 200 nM rapamycin (Rap) for 8 h. Strains were grown in liquid complete media (CM). NT = no treatment. RI = relative intensity calculated by normalizing Sch9 phosphorylation levels determined using anti-phospho-p70 S6 kinase antibody against p70 S6 kinase levels determined by anti-p70 S6 kinase antibody. Total proteins were separated on 10% SDS-PAGE, stained with Coomassie Brilliant Blue.

Since CgCFEM1 deletion led to a defect in appressorium formation (Figure 4), rapamycin (the specific TOR kinase inhibitor), cAMP and glutamine treatments were used to identify the potential signaling pathway by which CgCFEM1 functions. Figure 7 showed that cAMP and glutamine could not restore the appressorium formation rate of ∆*CgCFEM1* (Figure 7A,B), but rapamycin basically restored it (Figure 7C). Further, we examined the TOR activity by detecting the phosphorylation level of the p70-S6 kinase (S6K), a functional orthologue of yeast Sch9 and a TOR substrate demonstrated as a valuable tool to study the TOR activity. Immunoblot analysis showed the phosphorylation level of S6K was increased in the ∆*CgCFEM1* when compared with WT in the absence of rapamycin; however, in the presence of rapamycin, the TOR activity in WT was decreased by a third and that in ∆*CgCFEM1* was restored to a comparable level to that in WT in the absence of rapamycin (Figure 7D). These data inferred that the contribution of *CgCFEM1* in appressorium differentiation and formation was mediated by TOR signaling.

### 2.8. CgCFEM1 Suppressed Plant Immunity Responses

**Figure 8:**
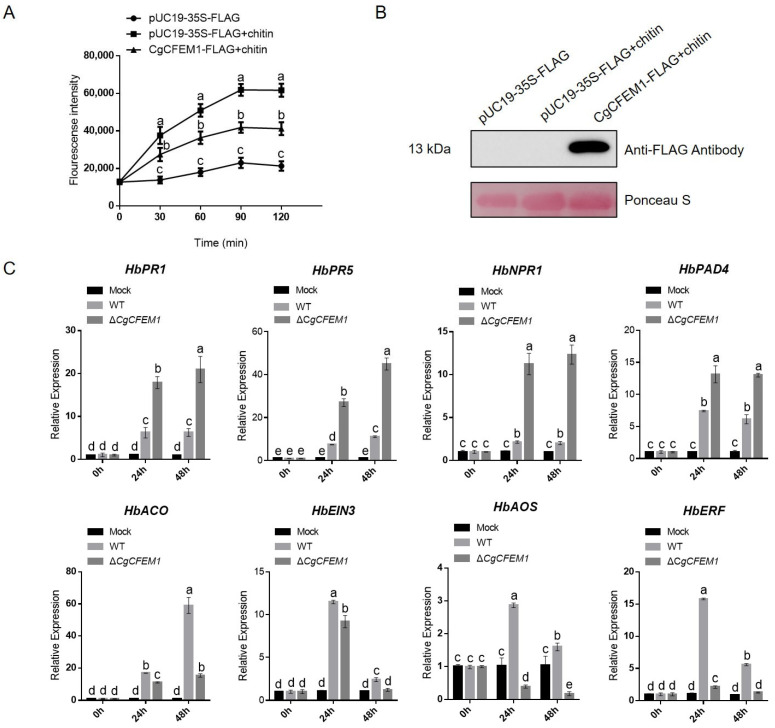
Effects of CgCFEM1 on ROS production and the expression of defense-related genes in rubber tree leaves. (**A**) Effects of CgCFEM1 on the ROS production induced by chitin. ROS productions were analyzed in rubber tree mesophyll protoplasts expressing empty vector without/with chitin treatment and expressing CgCFEM1 with chitin treatment. ROS contents were measured by DCFH2-DA. (**B**) Expression assay of CgCFEM1 in different samples. Expression level of CgCFEM1 was detected by immunoblot using anti-flag antibody. Ponseau S was used to detect protein loading. (**C**) Relative expression assay of defense-related genes in rubber tree leaves inoculated with WT, Δ*CgCFEM1*, and Res-Δ*CgCFEM1*. Data are shown as the means ± SD from three independent experiments. Different letters above columns indicate a significant difference (*p* < 0.05).

To evaluate the possible effects of CgCFEM1 on plant immunity, the chitin-triggered ROS production in rubber tree mesophyll protoplasts transiently expressing *CgCFEM1* was measured by DCFH2-DA (dichlorofluorescein diacetate) staining assay (Figure 8A,B). As shown in Figure 8A, in rubber tree mesophyll protoplasts expressing pUC19-35S-Flag vector, little change of ROS production was detected within 120 min, while in the same protoplasts treated with chitin, the ROS production increased sharply. When rubber tree mesophyll protoplasts expressing CgCFEM1-Flag fusion protein were treated with chitin, the ROS level was significantly lower than that of protoplasts expressing pUC19-35S-Flag vector with chitin treatment. These data indicated that CgCFEM1 suppressed chitin-triggered immunity.

In addition, the effect of CgCFEM1 on the expression of some defense-related genes such as *HbPR1*, *HbPR5*, *HbNPR1*, *HbPAD4*, *HbACO*, *HbEIN3*, *HbAOS* and *HbERF* in rubber trees was analyzed by qRT-PCR. As shown in Figure 8C, the expression of *HbPR1*, *HbPR5*, *HbNPR1* and *HbPAD4* significantly increased at 24 h and 48 h post-inoculation with ∆*CgCFEM1* compared to that inoculated with the WT strain. However, the expressions of *HbACO*, *HbEIN3*, *HbAOS* and *HbERF* were decreased significantly at 24 h and 48 h post-inoculation with ∆ *CgCFEM1* compared to that inoculated with WT. These results indicated that the CgCFEM1 probably impaired salicylic acid(SA)-mediated defense response but promoted ethylene (ET)- and jasmonic acid (JA)- mediated defense response in the rubber tree.

## 3. Discussion

Typically, CFEM proteins feature a signaling peptide, one or more CFEM domains and transmembrane domains or GPI anchor [26]. The CFEM proteins without transmembrane domains were defined as non-Pth11-like-type [6,7,8,9,10]. In our study, CgCFEM1 contained a conserved CFEM domain with 8 spaced cysteine residues (Figure 1A) and a signal peptide at N-terminal, but no transmembrane domains and GPI anchor (Figure 1B). Accordingly, CgCFEM1 structurally belonged to non-Pth11-like-type CFEM protein. Notably, some non-Pth11-like-type CFEM protein contained GPI anchor, while others did not, resulting in three branches in the phylogenetic tree being generated from identified fungal CFEM containing proteins including CgCFEM1 (Figure 1B). Taking this differentiation into consideration, we introduced a novel classification scheme encompassing three CFEM protein types. Type I featured solely a signal peptide and CFEM domain, Type II featured a signal peptide, CFEM domains and GPI anchor, while Type III featured a signal peptide, CFEM domain and transmembrane domains. Both Type I and Type II are consistent with the characteristics of secreted proteins acting as effectors [9,10]. Therefore, CgCFEM1 was a candidate effector.

Studies on several plant pathogenic fungi had demonstrated that CFEM proteins were involved in the regulation of fungal vegetative growth [13], conidial production, germination and consequent germ tube elongation [14,15], appressorium differentiation and morphogenesis [6,7,13], virulence [9] and infection process [26]. In our study, in addition to the reduced pathogenicity on rubber trees (Figure 3), CgCFEM1-deficient mutants also showed conidial morphological diversity (Figure 4A,B), reduced sporulation ability (Figure 4C), especially defective appressorium differentiation and primary invasion (Figure 4D–G), which was consistent with the expression pattern of CgCFEM1 at different development stages and during leaf infection (Figure 2A,B). These results indicated that CgCFEM1 contributed to virulence and played an important role in conidiation, spore morphogenesis, appressorium formation and primary invasion. In view of this, different CFEM proteins in various plant pathogenic fungi had diverse functions, which fully demonstrated the functional diversity of CFEM proteins in fungi.

Fungal appressorium development required the autophagic recycling of conidial cell contents [27,28]. Studies in *M. oryzae* had found appressorium morphogenesis a consequence of autophagy occurring within the spore after mitosis and nuclear migration [27]. In our study, we observed that type d conidia of ∆*CgCFEM1* contained three cells compared to type a conidia of WT with two cells (Figure 4A), Type II germ tubes (long germ tubes without the appressorium) and Type III germ tubes (long germ tubes with a dysplastic appressorium) of ∆*CgCFEM1* were composed of multicellular cells compared to Type I germ tubes of WT (normal germ tubes with single cell) (Figure 4B). Compared with WT that formed the mature appressorium, most germinating conidia of ∆*CgCFEM1* was unable to form an appressorium, except for a few that could form a dysplastic appressorium (Figure 4D,E). By MDC staining, we also observed obvious autophagosomes in germinating conidia of WT strain but not in ∆*CgCFEM1* (Figure 6). Taking these results together, CgCFEM1 was demonstrated to modulate conidiation, appressorium differentiation and morphogenesis through arresting mitosis rounds and autophagy.

The regulation of fungal appressorial formation was thought to be involved in three characterized signaling pathways, including cAMP/PKA, TOR (Target of Rapamycin) and glutamine signaling [29,30,31]. When conidia of the CgCFEM1 knockout mutant (∆*CgCFEM1*) were treated with rapamycin, cAMP and glutamine, respectively, in the process of appressoria formation induction, only rapamycin partially restored normal appressorial differentiation and formation, but not cAMP and glutamine (Figure 7A–C). This data indicated that the role of CgCFEM1 in appressorium formation was mediated by TOR signaling. In TOR signaling pathway, activated TOR kinase (TOR_on_) promoted cell growth and development, inactivate TOR kinase (TOR_off_) limited mitosis and induced autophagy and appressorium morphogenesis during spore germination [30,31]. Our data showed that the TOR activity in ∆*CgCFEM1* was always higher than that in WT (Figure 7D), indicating that CgCFEM1 might exert its influence on appressorium differentiation and formation by modulating TOR activity. Nonetheless, the mechanism by which CgCFEM1 regulates the TOR signaling pathway remains still unclear and warrants further investigation.

Some CFEM proteins had been reported to be involved in modulating host plant immunity, such as cell death, PAMP triggered ROS accumulation, callose deposition and defense-related genes expression [17,32] We previously had reported that transcriptional reprogramming of defense related genes *HbPR1* and *HbPR5* and ROS accumulation were induced in rubber tree mesophyll protoplasts treated with chitin [33]. To confirm the modulation of immunity by CgCFEM1, we investigated the effect of CgCFEM1 on ROS accumulation and the expression of defense-related genes. As expected, the presence of CgCFEM1 effectively suppressed chitin-triggered ROS production in rubber tree mesophyll protoplasts (Figure 8A), and it additionally influenced the expression of defense-related genes, including *HbPR1*, *HbPR5*, *HbNPR1*, *HbPAD4*, *HbACO*, *HbEIN3*, *HbAOS*, and *HbERF* in rubber tree leaves (Figure 8C). These data confirmed the potential role of CgCFEM1 in inhibiting chitin-mediated immunity, thereby facilitating invasion.

Phytohormones, as pivotal cellular signal molecules, played critical roles in mediating plant defense response against biotic and abiotic stresses [34]. Among these, salicylic acid (SA), jasmonic acid (JA), and ethylene (ET) were recognized as key players responsible for plant defense against biotic stress [35,36]. SA signaling was involved in PTI and ETI, inducing pathogenesis-related (PR) gene expression and increasing plant disease resistance [37]. Phytoalexin Deffcient4 (PAD4) contributed to SA accumulation [38,39]. 1-aminocyclopropane-1-carboxylic acid (ACC) synthase (ACS) and ACC oxidase (ACO) were two key enzymes in ET biosynthesis [40]. Transcription factors EIN3 and ERF were key regulators of ethylene signaling and important components of the ET signaling pathway, respectively [41,42]. Allene oxide synthase (AOS) was a major control point for JA biosynthesis [43]. In this study, the expression profiles of some genes involved in the SA, JA, ET defense response signaling pathway were analyzed in rubber tree leaves inoculated with WT and ∆CgCFEM1, respectively. The results showed that the genes involved in SA defense signaling (*HbPR1*, *HbPR5*, *HbNPR1*, and *HbPAD4*) were repressed significantly at 24 h in rubber tree leaves post-inoculation with WT compared to those inoculated with ∆CgCFEM1, but the genes involved in both JA and ET defense signaling (*HbAOS*, *HbERF*, *HbACO*, and *HbEIN3*) were increased in rubber tree leaves post-inoculation with WT compared to those inoculated with ∆*CgCFEM1*(Figure 8C). These data demonstrated that CgCFEM1 negatively regulated SA signaling, but positively regulated JA and ET mediated signaling pathway. It is well known that complex crosstalk between various hormone signaling pathways exists in plants; for example, antagonism between SA signaling and JA signaling, and synergistic interaction between JA signaling and ET signaling [40,41]. At present, we do not know yet whether this is caused by CgCFEM1 acting directly on these signaling pathways at the same time, or by one or two of them, and then through crosstalk. Therefore, it is worthwhile to further study the sophisticated regulatory mechanism of CgCFEM1 on plant hormone signaling in the future.

In conclusion, our study identified CgCFEM1 as a CFEM-containing effector of *C. gloeosporioides*, which contributed to the pathogenicity of *C. gloeosporioides* to rubber trees by manipulating the development of the invasion structure and inhibiting the defense response in the host plant.

## 4. Materials and Methods

### 4.1. Fungal Strains, Plants Materials, and Growth Conditions

*C*. *gloeosporioides* from *Hevea brasiliensis* (BioSample: SAMN17266943) wild type (WT) was used in this study. *C*. *gloeosporioides* strains were grown on potato dextrose agar (PDA) at 28 °C in the dark. *H*. *brasiliensis* (Reyan 7-33-97) plants were grown on soil in a growth room at 28 °C.

### 4.2. Bioinformatics Analysis

The amino acid sequence of *CgCFEM1* was deduced by DNAMAN 9.0 software. The CFEM domain-containing proteins from other fungi were retrieved from NCBI, and the CFEM domain sequences were extracted to perform a multiple sequence alignment by using Clustal W 1.81 and GeneDoc 2.7 software. The bootstrap neighbor-joining phylogenetic tree was constructed with MEGA 7.0 software and 1000 bootstrap repetitions were used in this method. Additionally, the domain architecture of the CFEM proteins was drawn with EvolView (https://www.evolgenius.info/evolview-v2/#login, accessed multiple times in October 2023), in this study. The NCBI Conserved Domain database was used to analyze the position of the CFEM domain. The GPI Modification Site Prediction (https://mendel.imp.ac.at/gpi/plant_server.html, accessed multiple times in October 2023), was used to analyze the potential GPI modification site. Prediction of the signal peptides and transmembrane helices was performed by SignalP 5.0 server and TMHMM 2.0 server, respectively.

### 4.3. Quantitative RT-PCR Analysis

To explore the expression pattern of *CgCFEM1*, samples from mycelia, conidia, appressoria and inoculated leaves were prepared as described previously [24]. For the RNA extraction from mycelia, conidial suspensions were inoculated into complete medium (CM) with an initial concentration of 1 × 10^3^ conidia mL^−1^ and incubated for 2 days at 28 °C under 120 rpm; then, mycelia were collected. As for the appressoria sample, a conidial suspension at a concentration of 1 × 10^5^ conidia mL^−1^ was plated onto a plastic plate and incubated at 28 °C for 24 h before the appressoria were collected with a cell scraper. For RNA extraction from the infection process towards the rubber tree, the conidial suspensions were sprayed onto the rubber tree leaves. The inoculated leaves were harvested at 0-, 1-, 2- and 3-days post-inoculation (dpi), respectively, and then frozen at −80 °C for total RNA extraction. The fungal RNA was extracted using TRIzol Reagent (Invitrogen, Waltham, MA, USA), as described [44]. As for plant total RNA extraction, the polysaccharide polyphenol plant total RNA extraction kit (TIANGEN Biotech, Beijing, China) was used according to the manufacturer’s instructions. Reverse transcription was conducted with FastKing gDNA Dispelling RT SuperMix (TIANGEN Biotech, Beijing, China). Quantitative RT-PCR analysis was performed with the QuantStudio™ 6 Flex Real-Time PCR Systems (Applied Biosystems, Foster City, CA, USA). The β-tubulin-1 (Cgβ-tub1) gene and 18S rRNA (Hb18S) were used as reference genes for normalization in *C. gloeosporioides* and in *H. brasiliensis*, respectively. All of the reactions had three biological replicates. The relative expression levels of the indicated genes were estimated on the basis of four technical replications using the 2^−ΔΔCt^ method.

### 4.4. Construction of CgCFEM1 Knockout and Complementary Strains

To generate the knockout mutant of *CgCFEM1*, the knockout replacement vector was constructed, as described [44], so that the 5′ (905 bp) and 3′ flanking regions (980 bp) of the *CgCFEM1* were amplified from genomic DNA of the WT strain with primer pairs. Vector pCB1532, carrying the acetolactate synthase gene (SUR) cassette conferring resistance to chlorimuron ethyl, was used as the backbone. The knockout vector was linearized with BamHI before transformation into protoplasts of the WT strain.

To generate the complementary strain, the complementary vector was constructed, as described [45], so that the nucleotide sequences of the *CgCFEM1* gene, together with its native promoter, were cloned and ligated the *HPT* (hygromycin phosphotransferase gene) cassette-containing vector pBARGPE1 under the manipulation of the trpC terminator. Then, the complementary vector was linearized before transformation into protoplasts of ∆*CgCFEM1*.

Protoplast preparation and transformation of *C. gloeosporioides* were performed, as described in our lab-established protocol [44]. The transformants were selected by resistance to chlorimuron ethyl or hygromycin B. As for the knockout mutants, the correct integration of the flanking regions into the target site of the genome was verified by conducting two independent PCR diagnoses with the primer pairs CgCFEM1-d5F/R and CgCFEM1-d3F/R, as shown in Figure S2. After that, the heterokaryon of the correct transformants were purified by single conidia isolation. Moreover, the full length of *CgCFEM1* in the knockout strains was amplified with that of the WT strain as a positive control. And the positive transformants of Res-Δ*CgCFEM1* were further confirmed through the PCR diagnosis of CgCFEM1 ORF.

### 4.5. Pathogenicity Assay

The pathogenicity assay was conducted as described in our previous work [25]. Briefly, droplets (5 µL, 2 × 10^5^ conidia mL^−1^) of the conidial suspensions were used to inoculate pre-wounded detached “light green” leaves from a rubber tree variety 73-3-97. The disease symptoms were scored at 4 days post-inoculation. Each treatment contained three replicates of 15 leaves and the entire experiment was repeated three times.

### 4.6. Fungal Growth and Conidiation Assay

For fungal growth assay, 5-mm-diameter hyphae agar disks were taken from the active colony edge and inoculated into minimal medium (MM) for 4 days, and the colony morphology and diameters were recorded. For conidiation assay, conidia were harvested from the strains growing on PDA medium for 7 days and inoculated into 50 mL liquid CM to the final concentration of 10^4^ conidia mL^−1^. The conidia numbers of indicated strains were recorded under a microscope after incubation at 28 °C with shaking (120 rpm) for 3 days. The experiments were repeated three times, with four replicates for each sample and ten microscope fields surveyed for every replicate.

### 4.7. Appressorium Development and Penetration Ability Assay

Appressorium development and invasion assay were performed by incubating indicated strains on plastic plates and onion epidermis, respectively, as described in our previous report [23]. Briefly, 5 drops (5 µL per drop, 2 × 10^5^ conidia mL^−1^) of conidial suspensions were placed on a plastic plate and incubated at 28 °C before the conidial morphology was observed, while the appressorium formation was observed under a microscope after 12 h incubation. Moreover, in order to identify the potential signaling pathway by which CgCFEM1 functions, the following treatments at the respective final concentrations were added to the conidial suspensions and analyzed at 24 hpi: 200 nM rapamycin (Rap; Beyotime Biotechnology, Shanghai, China), 10 mM 8-bromoadenosine 3′,5′-cyclic monophosphate sodium salt (8-Br-cAMP; Sparkjade, Jinan, China) and 10 mM L-glutamine (Macklin, Shanghai, China). All of the relative formation rates were calculated based on the data of three independent replicates, with at least 100 conidia per replicate.

### 4.8. Cell Staining

In order to stain the septa of the conidia, the counted conidia (1 × 10^5^ conidia mL^−1^) were dropped on a plastic plate for about 45 min and then the calcofluor white (CFW) dye solution was added to stain the samples for 10 min before they were observed under a fluorescence microscope with a UV laser. The conidial nuclei were stained with 5 μg mL ^−1^ DAPI staining for 3–5 min and observed in the same condition. As for autophagosome and autophagic vacuole staining, conidia placed on plastic plate for 0 h, 2 h, 4 h, 6 h were stained with 50 μM MDC for 30 min before they were observed under a fluorescence microscope with a fluorescence filter cube (excitation filter: 488 nm, emission filter: 505–550 nm). These experiments were repeated three times, with four replicates for each sample each time.

### 4.9. Immunoblot

To detect S6K/ Sch9 phospho-status, WT and Δ*CgCFEM1* were grown in CM, as above, and the mycelia were transferred to fresh CM with and without 200 nM Rap for 8 h. Mycelia harvested from the second growth regime were washed with distilled water three times and finely ground in liquid nitrogen. Equal amounts of mycelia powder were used for total protein extraction in a freshly prepared cell lysis buffer (60 mM Tris-HCl, pH 6.8, 2% SDS, 10% (*w*/*v*) glycerol, 5% β-mercaptoethanol) supplemented with protease inhibitors (5 mM EDTA, 1 mM PMSF, 1× cocktail) and phosphatase inhibitors (5 mM Na_3_VO_4_), followed by denaturation at 95 °C for 3 min. The cell lysates were cleared by centrifugation at 16,000× *g* for 15 min at 4 °C, and equal volumes of total proteins in lysates were resolved by 10% SDS-PAGE and then transferred to a PVDF membrane. Phosphorylation status of S6K/Sch9 was monitored using anti-phospho-p70 S6 kinase antibody (Beyotime Biotechnology, Shanghai, China) and normalized to p70 S6 kinase.

The blots were imaged using Clarity Western ECL chemiluminescent system (BioRad, Hercules, CA, USA) and quantitated by densitometry using ImageJ analysis software (https://imagej.net/Welcom, accessed multiple times in November 2023).

### 4.10. Reactive Oxygen Species Measurement

ROS measurement in rubber tree mesophyll protoplasm was carried out as previously reported in the protocol [33]. Mesophyll protoplasm expressing pUC19-35S-Flag and pUC19-35S-CgCFEM1-Flag were treated with or without 200 μg/mL chitin, respectively. ROS were measured every 30 for 120 min with the Reactive Oxygen Species Assay Kit (Beyotime Biotechnology, Shanghai, China), following the manufacturer’s instructions. Excitation/emission was 485 nm/530 nm for fluorescence reading with a fluorescence microplate reader (BioTEK, Winooski, VT, USA). This experiment was repeated three times. The mean fluorescence intensity of each treatment was obtained on the basis of four technical replications.

### 4.11. Statistical Analysis

Statistical significance analyses were performed using SPSS Statistics version 21.0. Data with a single variable were analyzed by one-way analysis of variance, and mean separations were performed by Duncan’s multiple range test. Differences at *p* < 0.05 were considered significant.

## Data Availability

Data are contained within the article and Supplementary Materials.

## Supplementary Materials

The following supporting information can be downloaded at: https://www.mdpi.com/article/10.3390/ijms25052937/s1.
