# Supplementary material for: CgCFEM1 Is Required for the Full Virulence of Colletotrichum gloeosporioides
Source: Int J Mol Sci. 2024 Mar 2;25(5):2937. doi: 10.3390/ijms25052937 (PMC10931812; doi:10.3390/ijms25052937)
Supplement: Supplementary file 1 [file ijms-25-02937-s001.zip › ijms-2875685-supplementary.pdf]

Supplementary Materials

1 ATGAAGACTTCCATCCTCGCCATCCTCGCTGTCGCCGCCGGCCTCGCTGTCGCCGACGAC

1 M K T S I L A I L A V A A G L A V A D D

61 ATCAAGCTGCCCGACTGCGCCAACGGCTGCCTCAACGACGGCCTCCAAAAGGCCGGCTGC

21 I K L P D C A N G C L N D G L Q K A G C

121 ACGCGAGACAACATCAAGGACTGCTACTGCCAGAAGGCCGACGTGAACTCTCTCGTCACC

41 T R D N I K D C Y C Q K A D V N S L V T

181 TGCGTCTCCAACAACTGCAAGAGCCAAAACGATCTGCTCGCGGCCGCACAATCCGCCGGC

61 C V S N N C K S Q N D L L A A A Q S A G

241 CAGATCTGCCCGAACGGCGTCGGCAACAACGGCCAGTTCGGCAACATCCCAGGCTTCCCC

81 Q I C P N G V G N N G Q F G N I P G F P

301 GGGAACAACAACAACAACAACGGCGGTGGCGGCCAGGGAGGTGGCCAGGGAGGCGGCAAC

101 G N N N N N N G G G G Q G G G Q G G G N

361 AACGGCGGTAACAAGGGCGGTAACTAA

121 N G G N K G G N *

Supplementary Figure S1 Nucleotide sequence and deduced amino acid sequence of CgCFEM1. Shading indicates the amino acid sequences of the signal peptide. Shading in grey indicates the region of the signal peptide, red font indicates the conserved cysteine residue, underline indicates the region of the CFEM domain, and the asterisk indicates the position of the stop codon.

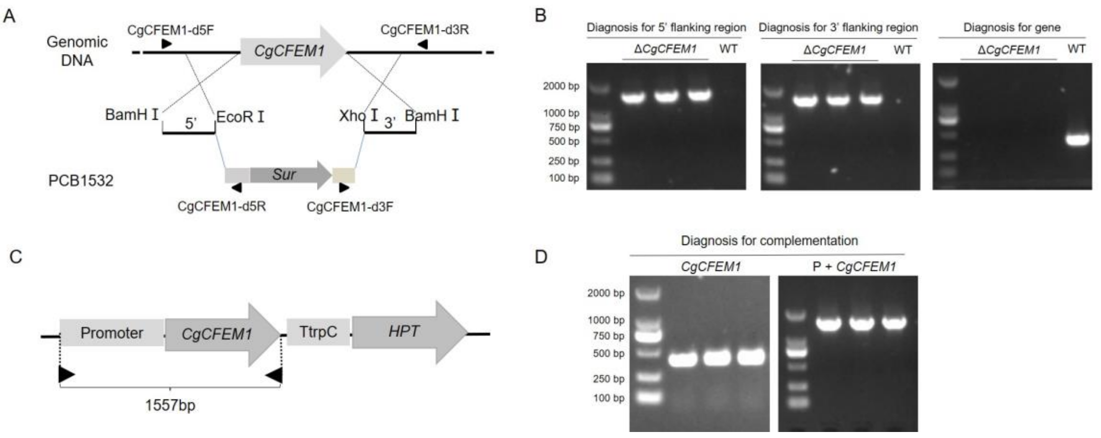

Supplementary Figure S2 Strategies and diagnosis for the knockout mutants and complementary mutants of *CgCFEM1*. (**A)** The schematic diagram of the *CgCFEM1* knockout. Primers of diagnosis are marked with black triangles. (**B)** Diagnosis for

flanking regions and CgCFEM1 nucleotide sequences in the ΔCgCFEM1 mutants; ΔCgCFEM1: the knockout mutants; WT: wild type. (**C)** The schematic diagram of CgCFEM1 complementation. (**D)** Diagnosis for CgCFEM1 nucleotide sequences (CgCFEM1) and their native promoter (P+ CgCFEM1). The DNA marker DL2000 was used for the fragment size analysis.

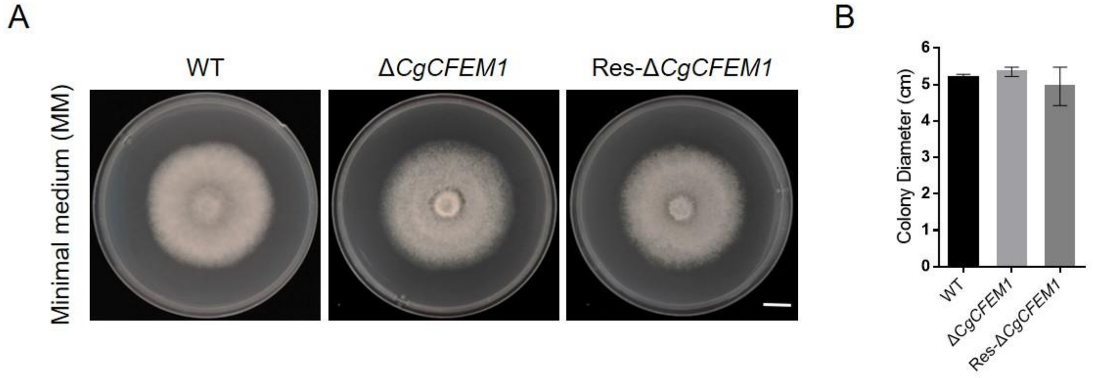

Supplementary Figure S3 Colony morphology and diameter of *C. gloeosporioides* strains cultured on minimal medium (MM). Scale Bar = 1 cm. **(A)** Colony morphology after growth on MM for 3 days. **(B)** Colony diameter after growth on MM for 3 days. Bars represent standard deviations (SD).
